# Dynamic mechanical analysis data of PEG/amorphous-silica composites

**DOI:** 10.1016/j.dib.2019.103731

**Published:** 2019-03-07

**Authors:** Allif Rosyidy Hilmi, Nur Aini Fauziyah, Gita Ayu Apriliyana, Suminar Pratapa

**Affiliations:** Department of Physics, Institut Teknologi Sepuluh Nopember (ITS), Keputih, Sukolilo, Surabaya, 60111, Indonesia

## Abstract

Data in this article presents the dynamic thermomechanical properties of PEG/amorphous-silica composites with different silica content, i.e. 0, 20 and 40% by weight. These composites were prepared using a solid-state method. The morphology of the amorphous-silica filler powder was determined using the transmission electron microscopes (TEM). Furthermore, the storage modulus (G′) data as a function of temperature were determined from the dynamic mechanical analysis (DMA) data in a shear mode. Moreover, the melting temperature and the activation energy for the degradation of each sample were also reported.

**Table:** Specifications table

Subject area	*Polymer and Composites.*
More specific subject area	*Characterization of Materials and Rheology.*
Type of data	*Table, image, text file and figure.*
How data was acquired	*Thermomechanical characterization (DMA/SDTA861, Mettler Toledo), transmission electron microscopy instrument (FEI Tecnai-T20).*
Data format	*Analyzed, plotted.*
Experimental factors	*During the formation of the composites, the PEG and amorphous silica solid powders were mixed and then the mixture was heated at 50°C, and subsequently cooled to room temperature to allow compaction.*
Experimental features	*DMA measurement was carried out in a shear mode from 25 °C to 80 °C, at a rate of* 5 °C/min*, at the frequencies of 1, 10, 100, 150 and* 200 Hz*, in a force of 0.1 N, at the displacement amplitude of* 100 μm*. The sample dimension was* 5 mm × 5 mm × 1 mm*.*
Data source location	*Advanced Material Laboratory, Department of Physics, Faculty of Science, Institut Teknologi Sepuluh Nopember, Surabaya, 60111, Indonesia.*
Data accessibility	*Data are available in this article* *URL:*https://data.mendeley.com/datasets/r84vsch36b/draft?a=7b9c8cc3-e73f-49db-8953-64363aa1dd4d
Related research article	*There are 2 articles that have been published and can be related to this article. Their DOIs are:*[1]https://doi.org/10.4028/www.scientific.net/AMR.1112.385 [2]*https://*doi*.org/*doi*.org/*10.1007*/*s12206*-*017-0703-2 *The data in this article is part of a research series of the above 2 papers but has not been published. The publication of the data in this article will, therefore, accomplish the whole data in the series.*

**Table:** 

**Value of the Data** • The DMA data of this composite with PEG as the matrix and a local amorphous silica as the filler can be used as a comparison with other commercial silica fillers in other polymer matrix composites (PMC). • The DMA data will be helpful to understand the effect of amorphous silica powder as a filler to the dynamic mechanical properties of the PEG-based composites as compared with other fillers particularly in our other PEG/silica composites which have been published previously. • The data can be used to understand for the relation between filler composition and activation energy for degradation of PEG/amorphous-silica composites.

## 1 Data

**Fig. 1:**
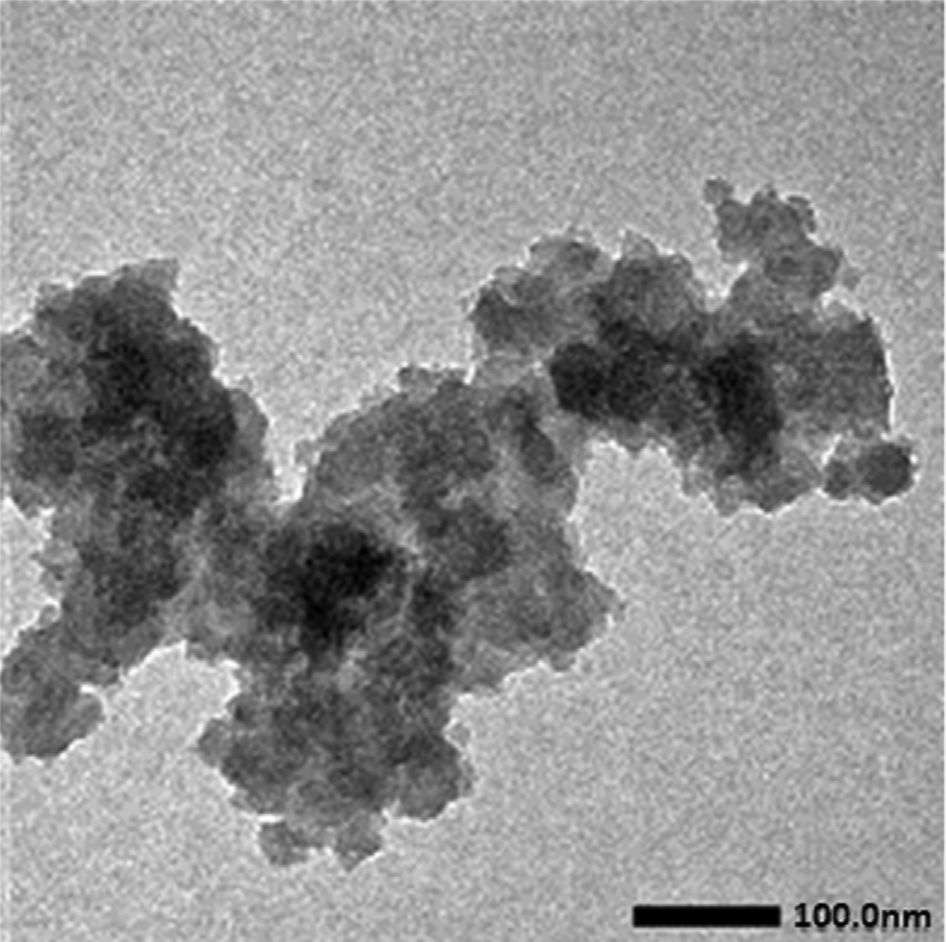
The TEM micrograph of the amorphous silica powders.

Data presented in this work describes the dynamic thermomechanical properties of PEG/amorphous-silica composites. In order to show the morphology of the amorphous silica filler about the characteristics of the composites, a transmission electron microscope (TEM) was used and the data is presented in Fig. 1.

**Fig. 2:**
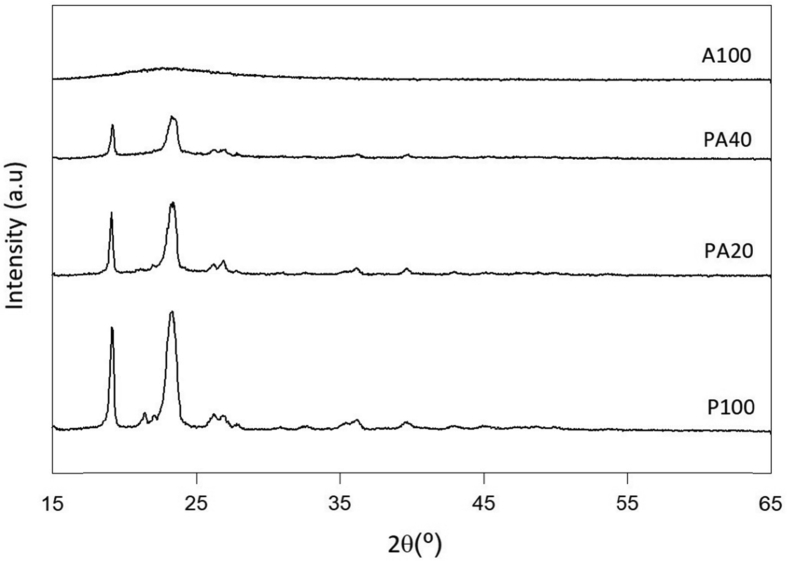
X-ray diffraction patterns (Cu-Kα radiation) of pure PEG (P100), pure amorphous silica (A100) and PEG/amorphous-silica composites (PA20 and PA40).

The composites were prepared by mixing with a solid-state method. The formation of the composites was confirmed using XRD patterns of the pure PEG and the composites as shown in Fig. 2.

**Fig. 3:**
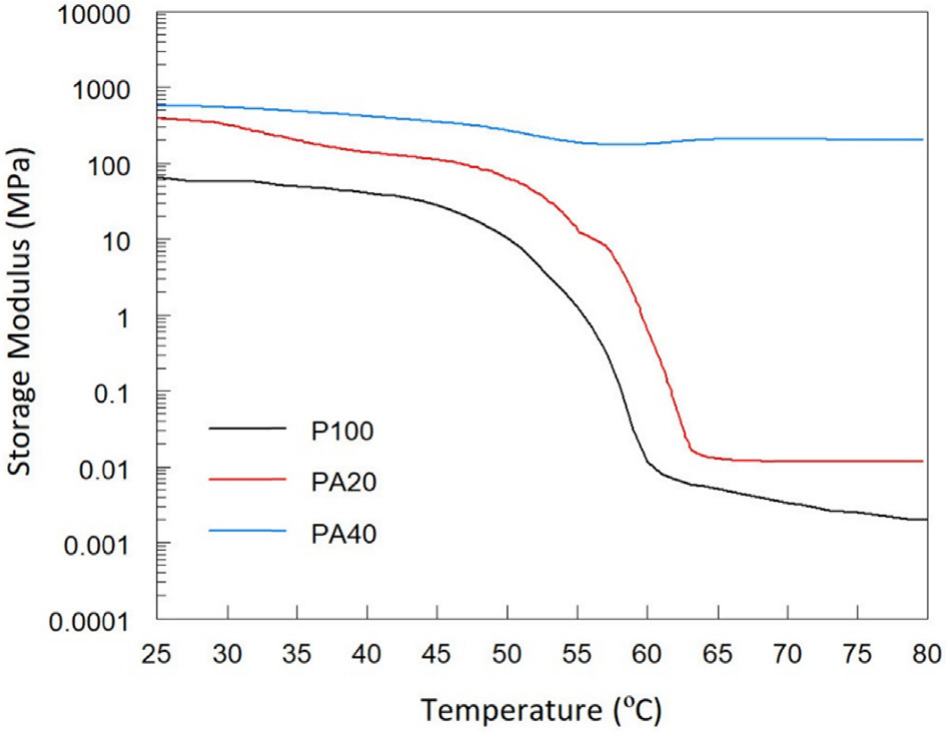
DMA shear storage modulus vs. Temperature for pure PEG and PEG/amorphous silica at 1 Hz.

The shear storage moduli data as a function of applied temperature for all specimens are presented. Fig. 3). These data were processed to determine the melting temperature and the activation energy for the degradation of PEG/silica composites which are presented in Table 1.

**Table 1:** Melting temperature and degradation activation energy ($E_{a}$) of pure PEG and PEG/silica composites.

Silica content (%wt.)	$T_{m}$ (°C)	$E_{a}$ (kJ/mol)
0	41.7	352
20	48.3	451
40	50.0	528

## 2 Experimental design, materials, and methods

The PEG/amorphous-silica composites with composition variation were prepared by using silica filler which was obtained from purified local silica sand. The sample preparation of the silica filler used in this data article was taken from a stage section in the recently related work [2].s

The composites were prepared using the solid-state method. The mixture of PEG and amorphous silica was heated at 50 °C then was cooled to room temperature to allow solidification. The filler content was 0, 20 and 40% amorphous SiO_2_ by weight, and thus the samples were designated as P100, PA20, and PA40, respectively.

**Fig. 4:**
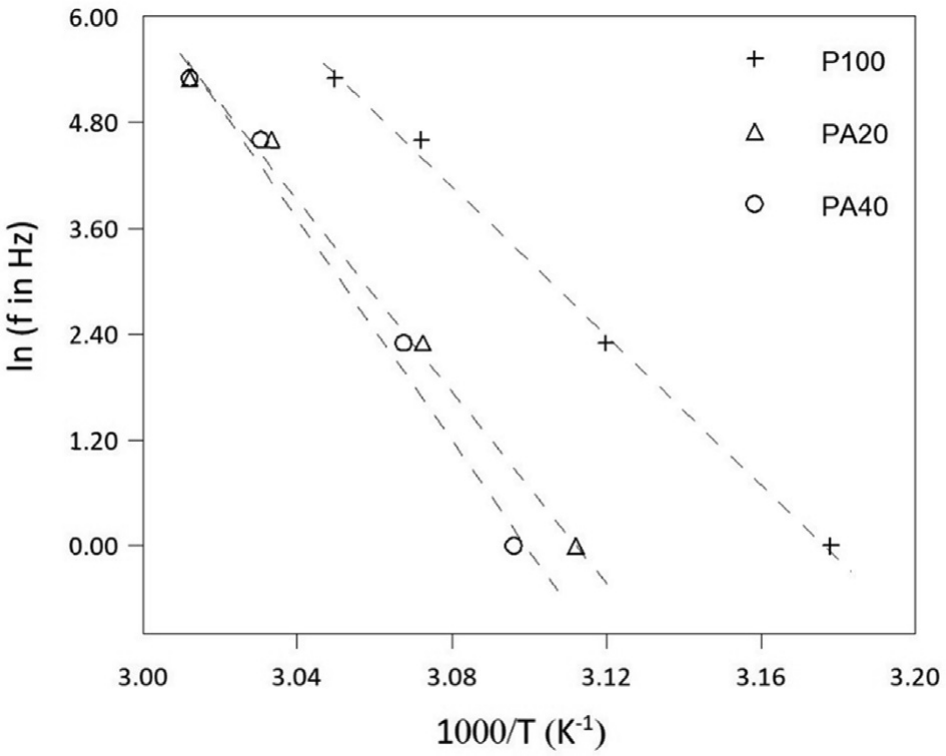
Plots of ln (*f* in Hz) against 1000/*T* according to the Arrhenius equation for the PEG/amorphous-silica composites.

The melting temperature is investigated by using the minimum value of the derivative of storage modulus, whereas the activation energy for degradation is determined from the Arrhenius equation

(1) $$f=f_{o}\;\exp\frac{-E_{a}}{RT}$$

where f is the applied frequency, T is the transition temperature (in this work, the transition temperature in this range temperature can be associated with the melting of the polymer, so that T = $T_{m}$*),* R is the universal gas constant, and $E_{a}$ is the activation energy for degradation. Here, we relate the degradation with the melting temperature for various applied frequencies. Then, for a particular sample, by plotting $T_{m}$ versus logarithmic frequency, a line will be obtained and from its slope, the activation energy for degradation of the sample can be gained. The plots for all samples are shown in Fig. 4.
