# Supplementary material for: Dynamic mechanical analysis data of PEG/amorphous-silica composites
Source: Data Brief. 2019 Mar 7;23:103731. doi: 10.1016/j.dib.2019.103731 (PMC6660471; doi:10.1016/j.dib.2019.103731)
Supplement: Supplementary file 1 — Multimedia Component 1 [file mmc1.doc]

Conflict of Interest and Authorship Conformation Form

Please check the following as appropriate:

- All authors have participated in (a) conception and design, or analysis and interpretation of the data; (b) drafting the article or revising it critically for important intellectual content; and (c) approval of the final version.
- This manuscript has not been submitted to, nor is under review at, another journal or other publishing venue.
- The authors have no affiliation with any organization with a direct or indirect financial interest in the subject matter discussed in the manuscript
- The following authors have affiliations with organizations with direct or indirect financial interest in the subject matter discussed in the manuscript:

Author’s name Affiliation

Allif Rosyidy Hilmi Department of Physics, Institut Teknologi Sepuluh Nopember. ITS. Keputih, Sukolilo, Surabaya 60111, Indonesia

Nur Aini Fauziyah As above

Gita Ayu Apriliyana As above

Suminar Pratapa As above
